# Designing phase 3 sepsis trials: application of learned experiences from critical care trials in acute heart failure

**DOI:** 10.1186/s40560-016-0151-6

**Published:** 2016-03-31

**Authors:** Alexandre Mebazaa, Pierre François Laterre, James A. Russell, Andreas Bergmann, Luciano Gattinoni, Etienne Gayat, Michael O. Harhay, Oliver Hartmann, Frauke Hein, Anne Louise Kjolbye, Matthieu Legrand, Roger J. Lewis, John C. Marshall, Gernot Marx, Peter Radermacher, Mathias Schroedter, Paul Scigalla, Wendy Gattis Stough, Joachim Struck, Greet Van den Berghe, Mehmet Birhan Yilmaz, Derek C. Angus

**Affiliations:** University Paris Diderot, Sorbonne Paris Cité, Paris, France; U942 Inserm, APHP, Paris, France; APHP, Department of Anesthesia and Critical Care, Hôpitaux Universitaires Saint Louis-Lariboisière, Paris, France; Department of Critical Care Medicine, St. Luc University Hospital, Université Catholique de Louvain (UCL), Brussels, Belgium; Center for Heart Lung Innovation and the Division of Critical Care Medicine, St. Paul’s Hospital, University of British Columbia, Vancouver, Canada; Adrenomed AG, Hennigsdorf, Germany; Università di Milano, Fondazione IRCCS Ca’ Granda, Ospedale Maggiore Policlinico, Milan, Italy; Département d’Anesthésie – Réanimation – SMUR, Hôpitaux Universitaires Saint Louis – Lariboisière, INSERM – UMR 942, Assistance Publique – Hôpitaux de Paris, Université Paris Diderot, Paris, France; Division of Epidemiology, Department of Biostatistics and Epidemiology, Perelman School of Medicine, University of Pennsylvania, Philadelphia, PA USA; Ferring Pharmaceuticals, Copenhagen, Denmark; Department of Anesthesiology, Critical Care and Burn Unit, St. Louis Hospital, University Paris 7 Denis Diderot, UMR-S942, Inserm, Paris, France; Department of Emergency Medicine, Harbor-UCLA Medical Center, Torrance, CA USA; Department of Surgery, Interdepartmental Division of Critical Care Medicine, University of Toronto, St. Michael’s Hospital, Toronto, Ontario Canada; Department of Intensive Care and Intermediate Care, University Hospital RWTH Aachen, Aachen, Germany; Institut für Anästhesiologische Pathophysiologie und Verfahrensentwicklung, Universitätsklinikum, Ulm, Germany; Campbell University College of Pharmacy and Health Sciences, Buies Creek, NC USA; Clinical Department and Laboratory of Intensive Care Medicine, Division of Cellular and Molecular Medicine, KU Leuven, Leuven, Belgium; Department of Cardiology, Cumhuriyet University Faculty of Medicine, Sivas, Turkey; CRISMA Center, Department of Critical Care Medicine, McGowan Institute for Regnerative Medicine, Clinical and Translational Science Institute, University of Pittsburgh Schools of the Health Sciences, Pittsburgh, PA USA; Department of Health Policy and Management, McGowan Institute for Regnerative Medicine, Clinical and Translational Science Institute, University of Pittsburgh Schools of the Health Sciences, Pittsburgh, PA USA

**Keywords:** Sepsis, Clinical trials as topic, Heart failure, Mortality, Multiple organ failure

## Abstract

Substantial attention and resources have been directed to improving outcomes of patients with critical illnesses, in particular sepsis, but all recent clinical trials testing various interventions or strategies have failed to detect a robust benefit on mortality. Acute heart failure is also a critical illness, and although the underlying etiologies differ, acute heart failure and sepsis are critical care illnesses that have a high mortality in which clinical trials have been difficult to conduct and have not yielded effective treatments. Both conditions represent a syndrome that is often difficult to define with a wide variation in patient characteristics, presentation, and standard management across institutions. Referring to past experiences and lessons learned in acute heart failure may be informative and help frame research in the area of sepsis. Academic heart failure investigators and industry have worked closely with regulators for many years to transition acute heart failure trials away from relying on dyspnea assessments and all-cause mortality as the primary measures of efficacy, and recent trials have been designed to assess novel clinical composite endpoints assessing organ dysfunction and mortality while still assessing all-cause mortality as a separate measure of safety. Applying the lessons learned in acute heart failure trials to severe sepsis and septic shock trials might be useful to advance the field. Novel endpoints beyond all-cause mortality should be considered for future sepsis trials.

**Electronic supplementary material:**

The online version of this article (doi:10.1186/s40560-016-0151-6) contains supplementary material, which is available to authorized users.

## Introduction

Sepsis, defined as "life-threatening organ dysfunction due to a dysregulated host response to infection" [1], is a major cause of mortality and morbidity worldwide [2–6]. Literature estimates of sepsis incidence vary widely [7]. One US study reported an absolute incidence ranging from 300 to 1031 cases per 100,000 population [7, 8]. The annual incidence of sepsis globally has been roughly estimated at 15 to 19 million [7, 9]. A systematic review of 33 studies originating in North America, Europe, Asia, and Australia found a population incidence for hospital-treated sepsis of 256 cases per 100,000 person-years [10]. The authors extrapolated these findings to estimate a global incidence for sepsis of 30.7 million cases, contributing to an estimated 6 million deaths each year [10].

Sepsis mortality has declined over the last decade from ~40 to ~20 % [11]. Improved processes of care (e.g., earlier diagnosis; timely resuscitation with appropriate therapies; low tidal volume during mechanical ventilation) may explain this observation [12–15]. However, neuromuscular, psychological, metabolic, cardiovascular, and renal complications persist and lead to impaired long-term outcomes among sepsis survivors [16, 17]. In addition, many sepsis patients are elderly and have other life-limiting comorbidities. Survival may be less important to these patients than measures reflecting independence and quality of life [18]. The long-term outcome and morbidity burden of sepsis survivors is an emerging, important research and clinical care concern.

Effective therapies are needed to better manage sepsis patients [19]. Therapeutic goals include not only improving survival but also reducing morbidity, preventing organ failure, and shortening convalescence [2, 20]. Substantial attention has been directed at reducing mortality in sepsis, but all recent multinational trials have failed to improve survival [21–26].

**Fig. 1:**
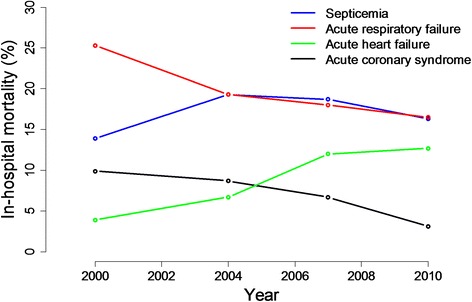
In-hospital mortality rates for septicemia, respiratory failure, and acute heart failure. Acute coronary syndrome included as an example of a critical care cardiovascular condition where reductions in in-hospital mortality have been realized. Rates are per 100 discharges for acute coronary syndrome, septicemia, and respiratory failure and were extracted from National Hospital Discharge Survey [66–68]. Rates for acute heart failure were based on published registry data [69] and represent percent of patients in the registries who died in the hospital. Data shown are from ADHERE [70] and OPTIMIZE [71] (2000), EHFS II (2004) [72], ALARM (2007) [73], AHEAD (2010) [74], and ATTEND (2011) [75]. The acute heart failure data should be interpreted considering the differences in registry populations and severity of illness

Other critical care conditions (e.g., acute heart failure) have faced similar challenges with efforts to prolong survival in clinical trials. Acute heart failure and sepsis are both critical care illnesses with high mortality. Both conditions represent syndromes with wide variation in patient characteristics, presentation, and standard management. Further, the underlying pathophysiology in both conditions is related to many processes, but pharmacologic interventions generally target single pathways and have not translated into survival benefits. All-cause mortality is usually the primary endpoint chosen for phase 3 pivotal trials in sepsis and acute heart failure, but no treatments to date have effectively reduced the high mortality associated with either of these conditions (Fig. 1). In a survey of acute heart failure experts, most felt it was unlikely that improvements in short-term mortality could be shown as a single primary endpoint in acute heart failure trials [27]. Thus, recent and ongoing acute heart failure trials have been designed with composite clinical primary endpoints, reserving all-cause mortality assessments for safety [28]. This approach recently adopted in some acute heart failure trials may help frame research in sepsis, since both of these critical care illnesses have faced similar challenges in clinical research.

The European Drug Development Hub brought together experts in critical care/sepsis and acute heart failure with the objective of sharing the collective clinical research experience in these critical care illnesses. The ultimate goal was to discuss better approaches to conducting clinical trials in critical care illnesses with high mortality (i.e., sepsis and acute heart failure) to promote advances in the care of these patients (Paris, France, January 2015). This paper summarizes the key developments from the meeting, focusing on clinical trial designs and endpoints that should be considered for use in future sepsis trials.

## Review

### Clinical trials in sepsis

#### Why have outcomes failed to improve in clinical trials?

An overview of the results from a selection of recent large, rigorously designed and conducted sepsis clinical trials reveals a consistent theme (Table 1). All trials were designed with short-term all-cause mortality as the primary endpoint, but none of the interventions has improved short-term survival for a variety of possible reasons (Table 2).

**Table 1:** Overview of key recent critical care sepsis trials

Trial	Design	Intervention	Study population	Mean SOFA^f^ score	Endpoint	Length of follow-up	*N* of deaths	Primary endpoint results
ALBIOS [22]	Multicenter, open-label, randomized, controlled	20 % albumin + crystalloid vs. crystalloid alone for 28 days or until ICU discharge	*N* = 1818 ≥18 years, clinical criteria for severe sepsis [76]	Albumin 8 (6–10) vs. crystalloid 8 (5–10)^a^, median (interquartile range)	All-cause mortality	28 days	285 albumin vs. 288 crystalloid	31.8 % albumin vs. 32 % crystalloid (RR 1.00, 95 % CI 0.87–1.14, *P* = 0.94)
SEPSISPAM [21]	Multicenter, open-label, randomized	Vasopressor treatment adjusted to maintain MAP of 80–85 mmHg (high target) vs. 65–70 mmHg (low target) for 5 days or until vasopressor support weaned	*N* = 776 Septic shock (Table 2) refractory to fluid resuscitation, requiring vasopressors	Low target 10.8 ± 3.1 vs. high target 10.7 ± 3.1^b^	All-cause mortality	28 days	142 high target vs. 132 low target	36.6 % high target vs. 34 % low target (HR for high target 1.07, 95 % CI 0.84–1.38, *P* = 0.57)
ProCESS [26]	Multicenter, randomized	Protocol-based EGDT vs. protocol-based standard therapy vs. usual care	*N* = 1341 Suspected sepsis with ≥2 criteria for SIRS, [76] and refractory hypotension or serum lactate ≥4 mmol/L	Not reported	All-cause in-hospital death	60 days	92 EGDT vs. 81 standard therapy vs. 86 usual care	21 % EGDT vs. 18.2 % standard therapy vs. 18.9 % usual care Combined protocol-based groups vs. usual care RR 1.04, 95 % CI 0.82–1.31, *P* = 0.83
Rosuvastatin for ARDS^e^ [25]	Multicenter, randomized, placebo-controlled, double-blind	Enteral rosuvastatin vs. placebo	*N* = 745 Positive pressure mechanical ventilation, PaO_2_ to F_IO2_ ratio ≤300, bilateral infiltrates on CXR without evidence of left atrial hypertension, known or suspected infection, and ≥1 criteria for SIRS (Table 2)	Not reported	All-cause mortality before hospital discharge home or until study day 60	60 days	108 rosuvastatin vs. 91 placebo	28.5 % rosuvastatin vs. 24.9 % placebo; difference 4.0 (−2.3 to 10.2), *P* = 0.21; enrollment stopped prematurely for futility
TRISS [23]	Multicenter, randomized, parallel-group	Leuko-reduced blood transfusion at lower (≤7 g/dL) vs. higher (≤9 g/dL) Hgb thresholds	*N* = 1000 ICU, fulfilled septic shock criteria (Table 2), Hgb ≤9 g/dL	Both groups 10 (8-12)^c^, median (interquartile range)	All-cause mortality	90 days	216 lower Hgb vs. 223 higher Hgb	43 % lower threshold vs. 45 % higher threshold (RR 0.94, 95 % CI 0.78 to 1.09, *P* = 0.44)
ARISE [24]	Multicenter, randomized, parallel-group	EGDT vs. usual care for 6 h	*N* = 1600 Suspected or confirmed infection, ≥2 criteria for SIRS (Table 2), refractory hypotension or hypoperfusion, identified in the ED within 6 h of presentation	Not reported	All-cause mortality	90 days	147 EGDT vs. 150 usual care	18.6 % EGDT vs. 18.8 % usual care (RR 0.98, 95 % CI 0.80 to 1.21, *P* = 0.9)
PROMISE [31]	Pragmatic, open, multicenter, parallel-group, randomized, controlled trial	6-h EGDT resuscitation protocol vs. usual care	*N* = 1260 Known or presumed infection, ≥2 SIRS criteria, and either refractory hypotension or hyperlactatemia within 6 h after ED presentation	EGDT 4.2 ± 2.4 vs. usual care 4.3 ± 2.4^d^	All-cause mortality	90 days	184 EGDT vs. 181 usual care	29.5 % EGDT vs. 29.2 % usual care (RR 1.01, 95 % CI 0.85 to 1.20, *P* = 0.9)

*ICU* intensive care unit, *MAP* mean arterial pressure, *EGDT* early goal-directed therapy, *SIRS* systemic inflammatory response syndrome, *CXR* chest radiography, *Hgb* hemoglobin, *ED* emergency department

^a^Includes subscores ranging from 0 to 4 for each of five components (respiratory, coagulation, liver, cardiovascular, and renal components), with higher scores indicating more severe organ dysfunction. The scoring was modified by excluding the assessment of cerebral failure (the Glasgow Coma Scale), which was not performed in these patients, and by decreasing to 65 mmHg the mean arterial pressure threshold for a cardiovascular subscore of 1, for consistency with the hemodynamic targets as defined according to the early goal-directed therapy

^b^Includes subscores ranging from 0 to 4 for each of five components (circulation, lungs, liver, kidneys, and coagulation). Aggregated scores range from 0 to 20, with higher scores indicating more severe organ failure

^c^Subscores ranging from 0 to 4 for each of six organ systems (cerebral, circulation, pulmonary, hepatic, renal, and coagulation). The aggregated score ranges from 0 to 24, with higher scores indicating more severe organ failure. One variable was missing for 51 patients in the higher-threshold group and for 64 in the lower-threshold group, so their values were not included

^d^Scores range from 0 to 24, with higher scores indicating a greater degree of organ failure. The SOFA score was calculated on the basis of the last recorded data before randomization. The SOFA renal score was based on the plasma creatinine level only and did not include urine output

^e^ *ARDS* acute respiratory distress syndrome

^f^ *SOFA* sequential organ failure assessment

**Table 2:** Reasons for lack of survival improvements in sepsis clinical trials

• Declining mortality rates over time
• Over-estimated treatment effects
• Suboptimal pre-clinical models
• Knowledge of pathophysiology is still evolving, making pathophysiologic targets difficult to identify
• Incorrect treatment targets
• Heterogeneity of the syndrome
• Heterogeneity of the patient population
• Improbability that a single treatment can impact key pathophysiologic processes that influence all-cause mortality

**Fig. 2:**
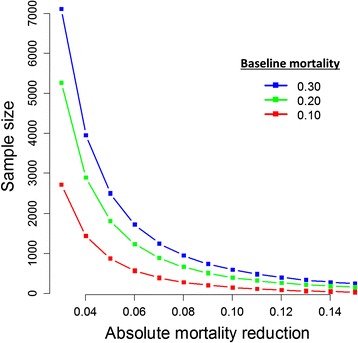
Estimated sample sizes by baseline mortality and absolute mortality reduction. This figure examines the total sample size needed to identify an absolute mortality reduction of 3 to 15 % assuming three control group mortality rates (30, 20, and 10 %). The assumptions in this figure is that power is 80 % for a two-sided test and that 1:1 randomization will be employed (for example, a total *N* of 3000 on the *y*-axis implies a *n* = 1500 in each treatment arm). Source: author calculations (MOH)

Mortality rates due to sepsis are declining but remain high [5]. Statistical power is dependent on several parameters, including the population’s baseline risk, the modifiable mortality, and on the treatment effect size and its variability within the study sample. In some recent sepsis trials, all-cause mortality ranged from 19 to 45 % depending on the study population and follow-up duration (Table 1). Achieving lower than expected event rates in trials (e.g., due to declining overall mortality, unintended enrollment of a lower-risk population, intentional exclusion of patients with an imminent risk of death) reduces the likelihood of identifying true treatment effects. As the overall mortality rate declines in the general sepsis population, the potential absolute effect of any given treatment is attenuated, if by nothing else, a lower fraction of modifiable mortality [19, 29]. At the same time, if baseline risk is higher than estimated, more patients will be needed as the expected treatment effect decreases (Fig. 2).

Over- or under-estimating treatment effects should be avoided when designing clinical trials [30]. Researchers have struggled and often over-estimated control group mortality when planning sample size and power estimates. For example, usual care group mortality rates were over-estimated by 5.1 % in Protocolized Care for Early Septic Shock (PROCESS) [26], 9.7 % in Sepsis and Mean Arterial Pressure (SEPSISPAM) [21], 19.4 % in Australasian Resuscitation in Sepsis Evaluation (ARISE) [24], and 10.8 % in Protocolized Management in Sepsis (ProMISE) [31], similar to previous over-estimates in septic shock trials (Vasopressin and Septic Shock Trial (VASST) over-estimate was 11 %) [32].

Sepsis is a complex syndrome characterized by the interplay of many pathways and systems. Sepsis therapies must either (1) control several pathways with several interventions or (2) hit “upstream” nodes that control a number of pathways. The treatment approach for sepsis has ranged from inhibiting the uncontrolled, inflammatory host response to enhancing the host immune response [33]. These seemingly conflicting approaches illustrate the complexity of the process and the significant (and ongoing) evolution in the understanding of sepsis pathophysiology. Analogously, the failure of positive inotropes to improve outcomes in clinical heart failure trials [34] was initially unexpected, but it was better understood as the knowledge of heart failure pathophysiology evolved.

Whether sepsis treatments targeting a single aspect of this complex syndrome could be reasonably expected to reduce all-cause mortality is uncertain. All-cause mortality is a robust endpoint because it reflects the net benefit of an intervention [28]. A benefit on all-cause mortality shows that the effect of the intervention is strong enough to overcome the influence of events on which the treatment has no or minimal effect [28]. While this approach works well when most deaths are directly related to the disease being studied, it may be less informative when heterogeneity in cause of death is common and mortality is often attributable to factors indirectly related to the disease such as occurs in sepsis [35].

In sepsis trials, significant patient heterogeneity exists in time to presentation and diagnosis, organisms(s), type and source of infection, organ involvement, degree of organ impairment, severity of illness, location of enrollment (e.g., emergency department vs. ICU), pre-existing conditions, and differences in standard of care across institutions or geographical regions (Additional file 1: Table S1) [36]. Recent consensus definitions for sepsis and septic shock should help to reduce this variation in future clinical trials (Additional file 1: Table S1) [1, 37]. The selection of sites participating in a clinical trial can substantially influence endpoints (e.g., variation in comorbidities or application of background therapies can impact event rates across high and low enrolling centers) and make interpretation of trial results difficult, a challenge that has been experienced in acute heart failure trials [38]. Genetic variants also appear to influence severity [39]. Treatment responses might vary, perhaps considerably, within such a group of patients according to clinical and genetic heterogeneity. Recent observational cohort studies highlighted the wide variation in mortality rates according to infection source [40]. At present, most trials do not consider heterogeneous treatment effects when estimating sample sizes. As a result, subgroup analyses, though often employed, are likely to miss important signals from treatments and interventions [19].

### Approaches to design clinical trials in sepsis

#### Characterization of pathophysiology: matching the treatment to the disease

Animal models used in sepsis do not accurately reflect the presentation of sepsis in humans [41, 42], in large part because there is no single presentation of sepsis in human disease. Validated and more clinically relevant animal models are needed to understand the disease process and enable therapy selection targeting specific pathophysiologic mechanisms. These models should replicate the duration of clinical intensive care treatment [42], integrate standard intensive care measures and advanced supportive care [42, 43], investigate higher order species to minimize the physiological and immunological differences between small animal species and humans [44–46], and investigate older animals with chronic comorbidities to better reflect real-world patient populations [42]. An alternate approach that might be more informative is to use the heterogeneity of animal models to understand predictors of treatment response, and then seek to replicate the predictors in a human trial. This approach has been explored in a systematic review of anti-tumor necrosis factor (anti-TNF) animal studies [47]. Biomarkers may play a role if they aid in diagnosis, prognosis (e.g., troponin in acute coronary syndrome [48] or N-terminal brain natriuretic peptide (NT-proBNP) in heart failure [49]), or identify patient subsets likely to respond to specific interventions (i.e., predictive biomarkers). Multi-biomarker approaches may be promising [50].

Although many advances in cardiovascular medicine were realized using the concept of large, simple trials, moving towards precision medicine has been proposed [51] (e.g., targeting patients with elevated systolic blood pressure for vasodilator trials in acute heart failure [52]). A similar approach has been suggested for sepsis trials, with emphasis on defining pathophysiology through better pre-clinical models, targeting drug development to specific pathophysiologic abnormalities, and selecting patients with clinical features likely to respond to a specific therapeutic approach or who are at sufficient risk for poor outcomes based on validated risk scores [42].

### Appropriate endpoints for sepsis clinical trials: insights from acute heart failure clinical trials

#### All-cause mortality

Reducing the morbidity burden in surviving patients is an important therapeutic goal that is not reflected in an all-cause mortality endpoint [53]. All-cause mortality is an appropriate endpoint when the population has a significant mortality risk and minimal competing risks and the intervention has the potential to alter the mortality risk. Short-term survival should predict longer-term survival with an acceptable quality of life. Sepsis satisfies the first criterion, but it performs poorly on the others. First, patients with sepsis die from many causes, but it is often impossible to determine which is primary (e.g., renal, hepatic, pulmonary, cardiac) [33]. Death occurs via many pathways, some of which are unrelated to the therapy being studied and will not be impacted by the treatment (e.g., a decision to withdraw support in many ICU cases [2]). The “noise” of non-response can obscure a beneficial effect on disease-specific death (i.e., the death that the intervention is able to impact). Thus, cause-specific mortality is a more informative endpoint to determine the benefit of a drug or intervention, whereas all-cause mortality is more meaningful when information on the net benefit of an intervention (i.e., benefit in the context of adverse events or non-response) is being sought [54]. In sepsis, cause-specific mortality is difficult to define but perhaps could be achieved in a clinical trial by increasing the “signal” (e.g., enrolling patients with the abnormality targeted by the intervention and exclude patients at low risk of death) and decreasing the “noise” (e.g., excluding patients with competing mortality risks from conditions unrelated to the sepsis episode). Cause-specific mortality might be useful in sepsis trials to identify agents with a significant treatment effect on specific components of the illness.

Similar to sepsis, patients with acute heart failure have high short-term mortality, a factor which usually makes mortality trials easier to conduct. However, in the case of acute heart failure, most therapies primarily target symptoms rather than the underlying pathophysiology that leads to death. Additionally, acute heart failure drugs are administered for a short-duration; both of these factors reduce the likelihood that all-cause mortality will be influenced over the intermediate or long-term (e.g., 180 days). Although the European Medicines Agency guideline still specifies all-cause mortality as the preferred primary endpoint in acute heart failure trials, it states that symptomatic improvement might be acceptable as a primary endpoint for short-term trials provided mortality is not adversely affected [55]. Regulatory agencies have recently agreed to a primary hierarchical clinical composite endpoint in an acute heart failure trial that combines a global assessment of symptoms, persistent or worsening heart failure requiring an intervention, and all-cause mortality assessed at 6, 24, and 48 h. Patients are categorized as improved (moderate or marked improvement in clinical status at all planned assessments without hospitalization for heart failure or death), unchanged (modest improvement or worsening in clinical status), or worsened (moderate or marked worsening of clinical status at any planned assessment, hospitalization for heart failure requiring intravenous or mechanical interventions, or death). The distribution of patients in each category is compared between treatment groups to assess the treatment effect [56, 57]. This endpoint has the advantage of reflecting considerations that are important to patients (both symptoms and outcomes), and it allows for a short-term assessment of morbidity and mortality during the period when the pharmacologic effect is present. Importantly, long-term all-cause mortality should still be assessed for safety, and the study should be powered to demonstrate that long-term mortality is not increased by a pre-specified safety margin [52].

Regulatory agencies might consider a similar clinical composite endpoint adapted for sepsis trials, where endpoints describing end-organ function, need for mechanical support, or need for other interventions are combined with short-term mortality (ideally sepsis-related mortality if consensus can be reached on a standard definition) as a primary endpoint, with longer-term all-cause mortality assessed for safety. This approach also has the advantage of reflecting relevant factors other than survival that are important to patients. Rigorous definitions for such endpoints are keys to ensure consistency and to reduce bias in the results and to ensure that the endpoint can be translated into a metric that is important to patients.

#### Non-fatal endpoints

Total or ICU length of stay has been considered as an endpoint for sepsis trials. It is relevant because ICU stays are costly, but it is dependent on external factors that are unrelated to drug therapy (e.g., physician judgment, no accepted standards for discharge readiness, availability of step-down beds, payer influence, local standards of care). These same limitations have been recognized in acute heart failure trials [58]. Thus, the length of stay is unsuitable as a primary endpoint for pivotal trials, but it can be useful as a secondary endpoint or to inform health technology and economic (cost/benefit) assessments. Other problems with using non-fatal endpoints include ascertainment bias, competing risks, and informative dropout when comparing treatment and control groups (i.e., patients who die cannot be hospitalized and patients who die early have decreased length of stay) [19].

Organ dysfunction is a relevant endpoint for sepsis trials. Multiple organs are impaired in sepsis [42], but all-cause mortality is insensitive to determine which organ or organ(s) are the primary driver of death. Conceptually, integrating a measure of organ dysfunction into a mortality endpoint (e.g., days alive and free of organ dysfunction) would provide a more comprehensive assessment of morbidity and mortality. Organ dysfunction is theoretically a more sensitive measure of the effect of an intervention on progression of the sepsis syndrome, but this concept has not yet been validated in trials. Since short-term organ dysfunction is associated with long-term outcome [17, 59], it is plausible that improvements in organ function might translate into improved survival, but this relationship has not yet been shown and the hypothesis still requires confirmation. The primary value of measuring organ dysfunction at the current time is to gain an understanding of how an intervention impacts physiology and organ function. Correlations between change in short-term organ dysfunction and long-term sepsis-associated morbidity could also be derived from large robust registries that include long-term follow-up and outcomes. If used as an endpoint, organ dysfunction should be pre-defined in the protocol and statistical analysis plan. Ideally, consensus about how to define organ dysfunction should be sought so that definitions are used consistently across clinical trials.

Days alive and free from mechanical ventilation, renal replacement therapy, or vasopressors (i.e., organ failure free days) has also been proposed. These endpoints are clinically meaningful, and widespread use of the Surviving Sepsis Campaign guidelines has led to more consistent timing and application of life support interventions. Nonetheless, the decision to institute supportive therapies is often subjective and can be influenced by external factors (e.g., reimbursement incentives, interactions of various medical specialists (e.g., intensivists and nephrologists)), which introduces increased variability (i.e., random noise) in the study and possibly bias if the study is not blinded. Other complex issues also warrant consideration, including whether patients value more event-free days equally regardless of when they occur (e.g., moving from 0 to 1 day is the same/better/worse than moving from 29 to 30 days), handling inclusion of multiple organs (i.e., are all organs of equal value or should failure in some organs be weighted more heavily than others), and methodology to account for pre-existing organ dysfunction. Interventions can be effective in *preventing* organ dysfunction (in patients who do not have organ dysfunction) and/or *preventing progression* of organ dysfunction (in patients who already have some degree of organ dysfunction). An adequate organ dysfunction scoring system must capture both of these possibilities.

In general, there are no accepted surrogates for safety [60], although death is not the only safety measure. Safety is difficult to assess in sepsis trials because of the high incidence of organ dysfunction in sepsis. Differences in organ dysfunction scores between treatment groups could also be seen as a safety outcome (e.g., prevention of organ dysfunction due to side effects of excessive vasopressor doses and duration). Other events (e.g., anaphylaxis) might be relevant for specific drugs. Even if a beneficial effect was shown on organ dysfunction or other non-fatal endpoint, adequate assurance of safety would still have to be demonstrated, either in a pivotal clinical trial, in the entirety of the drug’s database, or based on experience with similar drugs or interventions [60]. Consultation with regulatory agencies is needed to determine the size of the safety database and the confidence level required to rule out an adverse effect on mortality; these decisions are often dependent on the severity of illness in the population studied and the specific benefit of the drug (e.g., a drug that improves a clinically important outcome vs. a drug that improves control of a biomarker).

### Role of alternative study designs

#### Adaptive designs

Adaptive designs or seamless phase II/III designs have the potential to improve the efficiency of clinical trials. Adaptive designs can be particularly useful in fields in which data are limited to inform trial planning assumptions in the areas of expected event rates, anticipated effect sizes, heterogeneity of treatment effect, variance, safety, or drop-outs [61, 62]. In sepsis, many uncertainties exist at the time of trial design, and adaptive design is a promising approach for both exploratory and confirmatory stages of drug development, especially in the context of moving towards exploration of novel endpoints for sepsis trials. These designs are well accepted for feasibility and early phase studies, but as experience with their use has increased, they are becoming more accepted for pivotal trials as well [63]. Potential challenges include maintaining confidentiality and blinding of interim ongoing results and avoiding the introduction of bias resulting from the adaptations [63]. Strict control of type I error risk and understanding the potential biases are important issues; rapid progress is being made around these issues [64, 65].

Realistic trial simulation is the key tool to address these challenges and advance the field. Trial simulation of traditional and adaptive trial designs furthers understanding of strengths and weaknesses of proposed trial designs and will illustrate vulnerabilities from minor deviations to study design assumptions (e.g., event rates, missing data).

Ideally, trial design should be a multi-step, collaborative, and interactive multidisciplinary process between scientific, clinical, and statistical domain experts to increase the quality and chance of success. This concept applies to all types of trials, but it is particularly important for adaptive design. Early interaction with regulators is highly recommended when using adaptive designs in the later stages of a drug development program [61, 62].

## Conclusions

Sepsis is a major burden with high mortality, and the lack of progress in identifying effective treatments is discouraging for researchers and industry. The clinical research challenges that have been encountered in sepsis trials closely resemble those experienced by investigators in acute heart failure trials. After decades of research, it has become clear in the acute heart failure community that the substantial patient heterogeneity contributes to the difficulties in identifying effective therapies for the condition. The recent consensus definitions for sepsis and septic shock are important advances in this regard [1, 37].  Additionally, assessing all-cause mortality alone is insufficient to fully characterize the burden of disease because it omits important aspects of symptoms and functional status. Academic heart failure investigators and industry have worked closely with regulators for many years to transition acute heart failure trials away from relying on short-term symptoms and all-cause mortality as the primary efficacy measures, and ongoing trials are assessing novel clinical composite endpoints reflecting organ dysfunction and mortality while still evaluating all-cause mortality as a separate safety measure. Applying the lessons learned in acute heart failure trials to sepsis trials might be useful to advance the field (Table 3). Selecting high-risk patients with clinical phenotypes considered likely to respond to the intervention under study may help to reduce patient heterogeneity within clinical trials and enable signals of benefit to be more readily detected. Additionally, novel endpoints beyond all-cause mortality should be considered for future sepsis trials.

**Table 3:** Priorities for future sepsis clinical trials

1. Develop more informative studies using animal models
2. Emphasize study of pathophysiology
3. Identify biomarkers, molecular signals, or genetic markers to identify patients having an underlying causal process that might respond to the specific treatment being studied
4. Develop networks of sepsis investigators experienced in clinical trial conduct
5. Apply the recent Third International Consensus Definitions for Sepsis and Septic Shock [1, 37] when determining eligibility criteria
6. Conduct targeted clinical trials in relatively homogeneous groups of patients with characteristics suggestive of treatment response
7. Consider the addition of pre-specified covariate adjustment of the primary endpoint to address the issue of heterogeneity
8. Exclude low-risk patients if appropriate for the intervention being studied
9. Standardize care to reduce variability and random noise but not to the extent that results are not generalizable
10. Develop realistic expectations for treatment effect and power trials accordingly
11. Apply adaptive designs, especially when key variables are uncertain (e.g., event rates, expected treatment effect)
12. Consider targeted primary endpoints with all-cause mortality reserved for safety
13. Develop consensus in the field for standard trial definitions/criteria for interventions if used as endpoints (e.g., vasopressors, mechanical ventilation, renal replacement therapy)
14. Collaborate with regulators to modify approach to clinical trial design in this field
15. Develop robust registries to test external validity of the results of trials in broader patient populations
16. Discovery and development of a diagnostic that predicts a higher chance of response to a specific intervention

## Additional file

Additional file 1: Table S1. This table describes the definitions of sepsis and septic shock that have been used in pivotal sepsis trials. (DOCX 21 KB)

## Abbreviations

ADHERE: Acute Decompensated Heart Failure National Registry
AHEAD: Acute Heart Failure Database
ALARM: Acute Heart Failure Global Registry of Standard Treatment
ALBIOS: Albumin Italian Outcome Sepsis Study
Anti-TNF: anti-tumor necrosis factor
ARDS: acute respiratory distress syndrome
ARISE: Australasian Resuscitation in Sepsis Evaluation
ATTEND: Acute Decompensated Heart Failure Syndrome
CXR: chest radiography
ED: emergency department
EGDT: early goal-directed therapy
EHFS II: Second EuroHeart Failure Survey
Hgb: hemoglobin
ICU: intensive care unit
MAP: mean arterial pressure
NT-proBNP: N-terminal pro brain natriuretic peptide
OPTIMIZE: Organized Program to Initiate Lifesaving Treatment in Hospitalized Patients with Heart Failure
PaCO_2_: partial pressure of arterial carbon dioxide
PROCESS: Protocolized Care for Early Septic Shock
ProMISE: Protocolized Management in Sepsis
SEPSISPAM: Sepsis and Mean Arterial Pressure
SIRS: systemic inflammatory response syndrome
TRISS: Transfusion Requirements in Septic Shock
VASST: Vasopressin and Septic Shock Trial
