# Supplementary material for: Designing phase 3 sepsis trials: application of learned experiences from critical care trials in acute heart failure
Source: J Intensive Care. 2016 Mar 31;4:24. doi: 10.1186/s40560-016-0151-6 (PMC4815117; doi:10.1186/s40560-016-0151-6)
Supplement: Additional file 1: Table S1. — This table describes the definitions of sepsis and septic shock that have been used in pivotal sepsis trials. (DOCX 21 KB) [file 40560_2016_151_MOESM1_ESM.docx]

Supplementary Online Table. Definitions of sepsis and septic shock used in clinical trials

| Trial | Definition |
| --- | --- |
| ALBIOS [22] | Sepsis associated with organ dysfunction, hypoperfusion abnormality (e.g., lactic acidosis, oliguria, acute alterations of mental status), or sepsis-induced hypotension[76]  Fulfillment of each one of the following criteria:  1. Proved or suspected infection in at least one site:  a). Lung  b). Abdomen  c). Genitourinary tract  d). Other (blood, skin and soft tissue, central nervous system, bones and joints, cardiac system, catheter-related infection, other)  2. Two or more of the following:  a). A core temperature ≥38° C or ≤36° C;  b). A heart rate ≥90 beats/min;  c). A respiratory rate ≥20 breaths/min or ^a^PaCO2 ≤ 32 mmHg or use of mechanical ventilation for an acute process;  d). A white blood cell count ≥12000/ml or ≤4000/ml or immature neutrophils >10%.  3. Presence of at least a severe and acute sepsis-related organ dysfunction, as measured by the modified Sequential Organ Failure Assessment (SOFA) score:  a). Respiratory score >1;  b). Hematologic score >1;  c). Hepatic score >1;  d). Cardiovascular score equal to 1, 3 or 4;  e). Renal score >1. |
| SEPSISPAM [21] | Presence of ≥2 diagnostic criteria of the systemic inflammatory response syndrome, proven or suspected infection, and sudden dysfunction of ≥1 organ[76] |
| ProCESS [26] | Presence of ≥2 of the following 4 criteria:  1. Temperature >38°C or <36°C;  2. Heart rate >90 beats per minute  3. Respiratory rate >20 breaths per minute or PaCO_2_ <32 mmHg  4. White blood cell count >12,000/mm^3^, <4,000/mm^3^ or >10% immature (band) forms |
| Rosuvastatin for ARDS [25] | Either of the following SIRS^b^ criteria:  1. White cell count >12,000/mm^3^ or <4,000/mm^3^ or differential count with >10% band forms  2. Core body temperature >38°C or <36°C |
| TRISS [23] | Presence of ≥2 SIRS criteria:  1. Core temperature >38°C or <36°C  2. Heart rate ≥90 beats per minute  3. Mechanical ventilation for an acute process, or respiratory rate ≥20 breaths per minute, or a PaCO_2_<4.3 kPa (32 mmHg)  4. White blood cell count >12,000/mm^3^ or <4,000/mm^3^  And  Suspected or verified focus of infection (organism grown in blood or sterile site or abscess/infected tissue)  And  Hypotension (SBP^c^ ≤90 mmHg or MAP^d^ ≤70 mmHg) despite fluid therapy or vasopressor/inotrope infusion to maintain blood pressure |
| ARISE [24] | Presence of ≥2 of the following 4 criteria:  1. Temperature >38°C or <36°C;  2. Heart rate >90 beats per minute  3. Respiratory rate >20 breaths per minute or PaCO_2_ <32 mmHg or the requirement for invasive mechanical ventilation for an acute process  4. White blood cell count >12,000/mm^3^, <4,000/mm^3^ or >10% immature (band) forms  And  Refractory hypotension (SBP <90 mmHg or MAP <65 mmHg) after an IV fluid challenge of ≥1000 mL administered within 60 minutes; or hypoperfusion defined as blood lactate ≥4 mmol/L |
| Sepsis-3 [1,37] | Third International Consensus Definition for Sepsis: Life-threatening organ dysfunction due to a dysregulated host response to infection. Recommended criteria for sepsis are a SOFA^e^ score of ≥2 points in encounters with suspected infection in the ICU^f^, and use of qSOFA^g^ in non-ICU settings to consider the possibility of sepsis  Third International Consensus Definition for Sepsis Shock: A subset of sepsis in which underlying circulatory, cellular, and metabolic abnormalities are associated with a greater risk of mortality than sepsis alone. Adult patients with septic shock can be identified using the clinical criteria of hypotension requiring the use of vasopressors to maintain mean blood pressure of 65 mmHg or greater and having a serum lactate level greater than 2 mmol/L persisting after adequate fluid resuscitation. |

^a^PaCO_2_ = partial pressure of arterial carbon dioxide; ^b^SIRS = systemic inflammatory response syndrome; ^c^SBP = systolic blood pressure; ^d^MAP = mean arterial pressure; ^e^SOFA = Sequential (sepsis-related) Organ Failure Assessment. SOFA score ranges from 0 to 24 where 0 to 4 points are assigned for 1 of 6 organ dysfunctions: hematologic, hepatic, respiratory, neurologic cardiac, and renal. A greater score corresponds to greater severity. Maximum score is determined from 48 hours before to 24 hours after onset of infection; ^f^ICU =intensive care unit; ^g^qSOFA = quick Sequential (sepsis-related) Organ Function Assessment. Final qSOFA model included Glasgow Coma Scale score ≤13, systolic blood pressure ≤100 mmHg, and respiratory rate ≥22 per minute (1 point each, score range 0-3).
